# Ultrafast and efficient continuous flow organic synthesis with a modified extruder-grinder system

**DOI:** 10.1038/s41598-024-59567-6

**Published:** 2024-04-27

**Authors:** Omid Hosseinchi Qareaghaj, Mohammad Ghaffarzadeh, Najmedin Azizi

**Affiliations:** Chemistry and Chemical Engineering Research Center of Iran, P.O. Box 14335-186, Tehran, Iran

**Keywords:** Extruder-grinder, Continuous system, Catalyst-free, Solvent-free, Multicomponent synthesis, Chromenes, Green chemistry, Environmental sciences, Chemistry

## Abstract

The study introduces a groundbreaking continuous system that combines an extruder and grinder to enable catalyst-free and solvent-free reactions under mild conditions. This temperature-controlled system facilitates the synthesis of highly functionalized chromenes, which have valuable applications in generating combinatorial libraries and complex target molecules. The newly developed mill extruder machine offers several advantages for industrial production on a large scale. It effectively reduces waste, saves energy, and enhances time efficiency. This system represents a significant advancement in the field, providing a new strategy for one-pot synthesis of various types of highly functionalized spirooxindoles and chromenes. Remarkably, these reactions can be accomplished within a short timeframe of 2–10 min, yielding impressive results of 75–98%. The results demonstrate superior performance compared to traditional reaction methods, making it an appealing tool and hotspot area of research in green chemistry.

## Introduction

Sustainability is playing an increasingly important role in environmental and industrial processes, and the expertise of sustainability professionals is essential in addressing the associated challenges. Solvents and catalysts are critical elements in numerous chemical processes, but their use can lead to environmental pollution due to the presence of hazardous chemicals^1^. Specifically, the large quantities of volatile, flammable, and toxic organic solvents, as well as metal catalysts, contribute significantly to this issue^2^. In recent years sustainable alternatives solvents and catalysts are being developed and implemented. Green solvents, eco-friendly catalysts and novel tools are being explored to minimize the environmental impact of chemical processes^3^.

Mechanochemistry has garnered significant interest among organic chemists as a versatile technique for conducting rapid, clean, and environmentally friendly synthesis without the need for harmful organic solvents^4^. This approach involves utilizing mechanical forces such as shearing, grinding, or milling to facilitate chemical reactions. Ball milling mechanochemistry, in particular, has proven to be a widely applicable method in solid-state phase reactions, offering advantages over conventional laboratory work involving stirrers and heaters^5^. Large-scale ball mills are utilized mainly for material processing but are not yet employed efficiently in organic chemistry^6^. Ball milling has indeed brought about a revolution in organic synthesis by enabling faster and simpler reactions in a solvent-free environment, leading to high conversion rates. However, there are two key areas that require attention and improvement in these methods: temperature control and scalability. Addressing these deficiencies is crucial given the significance of mechanical processes in organic synthesis^6^.

**Figure 1:**
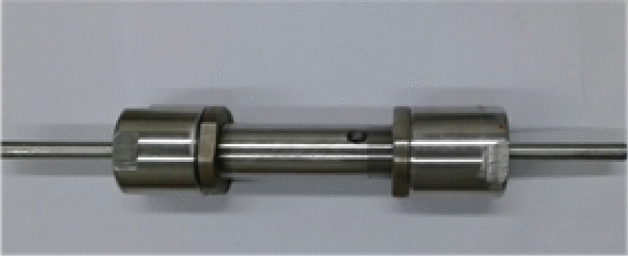
Twin-screw extrusion.

To eliminate these restrictions, scientists have introduced a twin-screw extrusion (TSE) (Fig. 1) in organic chemistry. Reactive extrusion is an innovative and efficient technique used in the synthesis of polymers. It involves combining polymerization or chemical reactions with the process of extrusion to produce functionalized or modified polymers^7^. This method offers several advantages over traditional polymer synthesis approaches, including improved reaction kinetics, enhanced control over the polymer structure, and simplified process integration. Furthermore, twin-screw extrusion has found applications beyond co-crystal synthesis, including the synthesis of diverse chemical species such as metal–organic frameworks (MOFs) and, deep eutectic solvents^8^.

**Figure 2:**
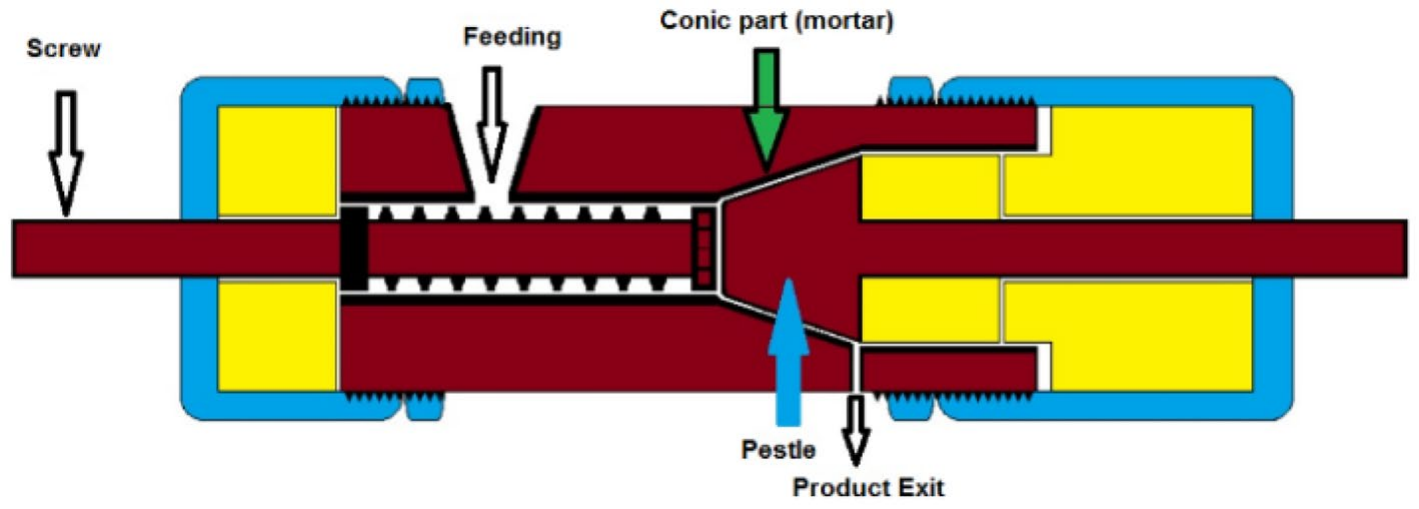
Reactive continuous extruder-milling (REG).

This study introduces reactive extruder-grinder (REG) as a new technique in continuous mechanochemical organic syntheses for the first time. Figure 2 shows the designed REG device, which is a combination of an extruder, a mortar, and a pestle. The REG operates in a continuous manner for mechanochemical organic syntheses. The materials are fed into the REG and mixed thoroughly within the extruder. They are then pushed into the conic part of the device. REG is capable of continuous reactive synthesizing of organic compounds in quantities ranging from milligrams to several grams and above. At the end of the cylinder, the conic part is designed to play the role of the mortar, and the final process of material grinding and heat-generating occurs in it. The conic part is the most critical part of the REG. By grinding the materials between the conic part wall and the pestle, the required heat is generated. The chemical reaction is carried out quickly due to the intensive grinding and generated heat. In REG, the screw and the pestle are designed in a single piece and alignment. The connection and coherence of these two parts have made the rotation and feeding uniform. The ability to continually enter the raw material and the regular withdrawal of the reaction product has made it an efficient device that can perform various condensation reactions quickly. The materials become mills intensively, and the reaction temperature increase due to the friction. The generated temperature can effectively cause the fulfillment of the multicomponent reactions (MCRs) that require heat. The twin-axis rotational speed of the screw and pestle was adjusted to control the reaction temperature by empirically controlling the screw and pestle rotational speed. The ability to continually enter the raw material and the regular withdrawal of the reaction product has made it an efficient device that can perform various condensation reactions quickly.

Amino-4H-chromenes are versatile organic compounds characterized by a chromene ring system with an attached amino group^9^. These compounds exhibit a range of biological activities, including antioxidant, anticancer, anti-inflammatory, antimicrobial, and potential antiviral effects^10^. Their unique chemical structure and diverse activities make them attractive candidates for drug discovery and development^11^. With their potential to modulate various disease pathways, amino-4H-chromenes hold promise for the development of novel therapeutics targeting conditions such as cancer, inflammation, and infectious diseases^12^. Continued research in this field aims to uncover their full therapeutic potential and optimize their pharmacological properties for clinical applications^13^.

The indole ring system, known for its biologically active derivatives, plays a crucial role in the structure of numerous pharmaceutical molecules^14^. Among these derivatives, spirooxindole compounds have gained recognition in diverse fields, particularly as inhibitors of microtubule assembly. Compounds such as spirotryprostatin A and B have shown potential in inhibiting microtubule assembly^15^. Additionally, spirooxindole derivatives have demonstrated their ability to modulate Muscarinic M1 and serotonin receptors, exemplified by compounds like pteropodine and isopteropodine^16^. Furthermore, spirooxindole compounds, including MK-0677, exhibit promise as nonpeptidyl growth-hormone secretagogues^17^. Given the significance of spiro compounds in organic chemistry and pharmacology, several synthetic protocols have been reported, and ongoing research efforts are exploring this subject^18^. The continued exploration of spirooxindole derivatives holds potential for the development of novel therapeutic agents and the advancement of organic chemistry.

Knoevenagel condensation of malononitrile and chlorobenzaldehyde at room temperature, with only around 7% of the desired product obtained after 10 min in the solid state^19^. However, it has been demonstrated that conducting the reaction through a melting process at 150 °C without a catalyst can yield quantitative results. Stoichiometric Michael additions using dimedone at temperatures ranging from 100 to 130 °C have shown complete formation of 7,7-dimethyl-5-oxo-5,6,7,8-tetrahydro-4H-chromenes in quantitative yields^19^. Furthermore, it was shown that incorporating a mild catalyst (Na_2_CO_3_) during the milling process at ambient temperature led to quantitative yields of diverse chromene derivatives. The inclusion of Na_2_CO_3_ during kneading optimizes the overall efficiency of Knoevenagel condensations and Michael addition reactions, ensuring complete conversion. These findings underscore the significance of reaction conditions and catalysts in achieving high yields and complete conversion in the synthesis of chromene derivatives.

**Figure 3:**
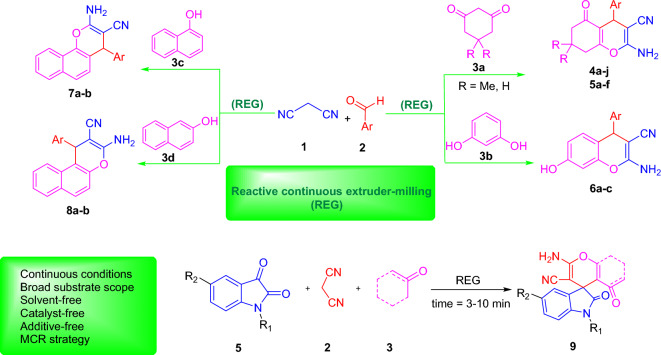
A schematic representation of prepared products in REG device.

In our ongoing pursuit of environmentally friendly organic transformations^20^, we present a groundbreaking study introducing the REG device as a reactive reactor for the solvent-free, catalyst-free, and additive-free multicomponent synthesis of diverse pyran, 2-amino-4-aryl-3-cyano-4H-chromene, and spiroxindole derivatives. Importantly, this innovative method boasts the advantage of not relying on additives or catalysts, while ensuring remarkably short reaction times. The findings of this investigation were illustrated and presented in Fig. 3.

## Results and discussion

To optimize the reaction parameters, a one-pot, multicomponent reaction (MCR) was performed involving 4-chlorobenzaldehyde (1a), malononitrile (2), and dimedone (3a) as the model reaction. The utilization of the REG device enabled the execution of a multicomponent reaction without the requirement of an external trigger. This innovative device facilitated the reaction to proceed spontaneously, eliminating the need for additional stimuli or initiators. Inside the REG device, the raw materials are initially mixed in the screw section, resulting in a uniform mixture that then enters the mortar section. In this section, the intensive friction and controlled rotation speed generate heat, which plays a crucial role in facilitating the cascade reaction of Knoevenagel condensation and Michael addition. The combination of intensive grinding and heat assistance within the REG device ensures the efficient and quantitative formation of the desired product.

**Figure 4:**
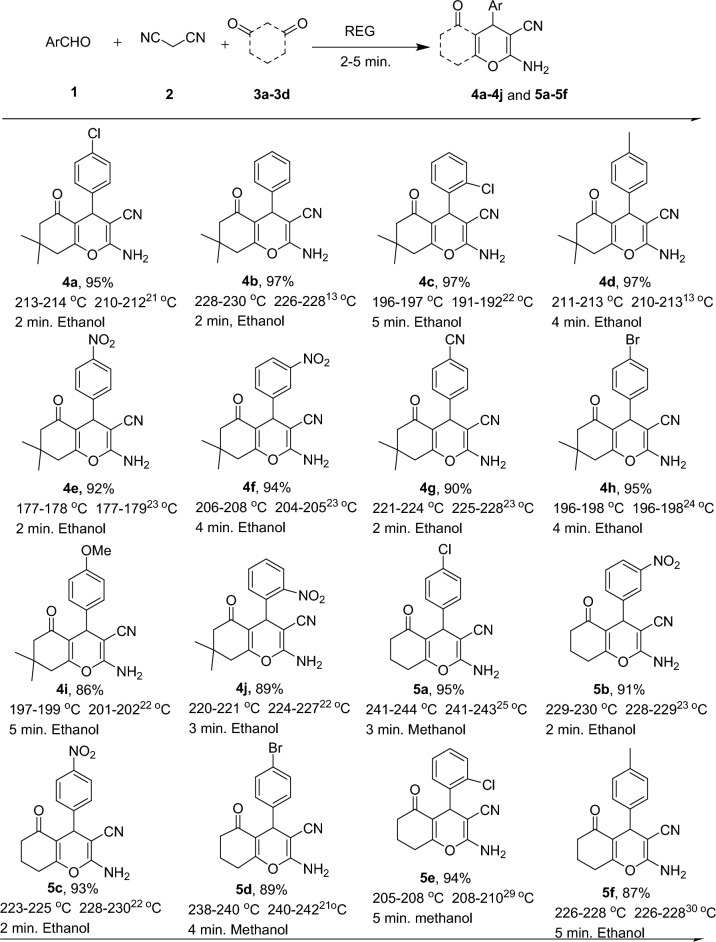
Synthesis of various 4*H*-chromene in REG in optimized reaction conditions. ^a^Isolated pure product. ^b^Recrystallization solvents.

Figure 4 presents the results obtained under optimized conditions for the multicomponent reaction involving various aldehyde derivatives and malononitrile with different reactants, including dimedone (3a) and 1,3-cyclohexanedione (3b), The table showcases that a wide range of aldehydes, encompassing both electron-donating and electron-withdrawing groups, yielded excellent to good yields within a remarkably short reaction time of 2–5 min. These findings demonstrate the efficiency and versatility of the reaction system in synthesizing the desired products using different aldehyde derivatives and reactants.

**Figure 5:**
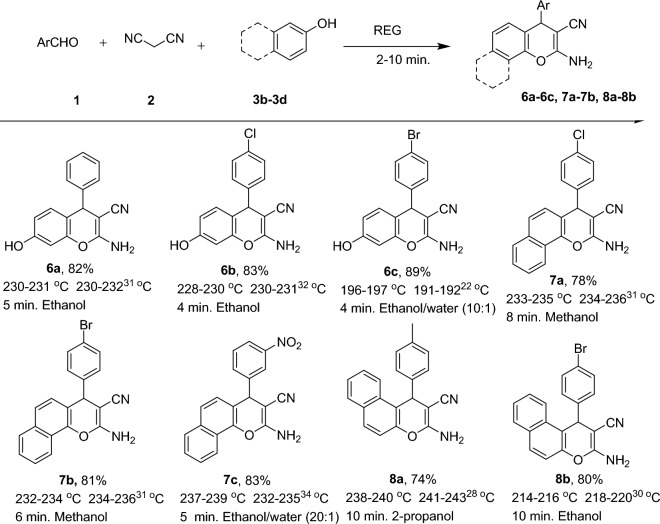
Synthesis of various 4*H*-chromene in REG in optimized reaction conditions. ^a^Isolated pure product. ^b^Recrystallization solvents.

The scope and versatility of a one-pot reaction in REG were extended to activated phenol derivatives such as resorcinol (3c), α-naphthol (3d), and β-naphthol (3c). The results of these reactions are presented in Fig. 5. All activated phenol compounds were found to be good participants in this system, yielding good to excellent yields in short reaction times. The use of REG enabled ultrafast reactions to be conducted at ambient temperature without the need for any catalyst or additive on a 5 mmol scale. The experimental procedure was described as straightforward and easy. Initially, the raw materials were pre-mixed in a traditional mortar and pestle. The mixed materials then entered the first part of the REG machine, referred to as the screw, which featured a high homogeneous mixing rate. Subsequently, the well-mixed materials entered the mortar section of the REG system.

Spiroxindole and its derivatives are considered privileged heterocyclic cores due to their presence in many natural products and pharmacological agents^21–24^. These compounds have found extensive use in the pharmaceutical industry due to their versatile role in numerous biologically active substances^25–27^. These characteristics have made them highly valuable in the development of novel pharmaceutical agents^28^.

**Figure 6:**
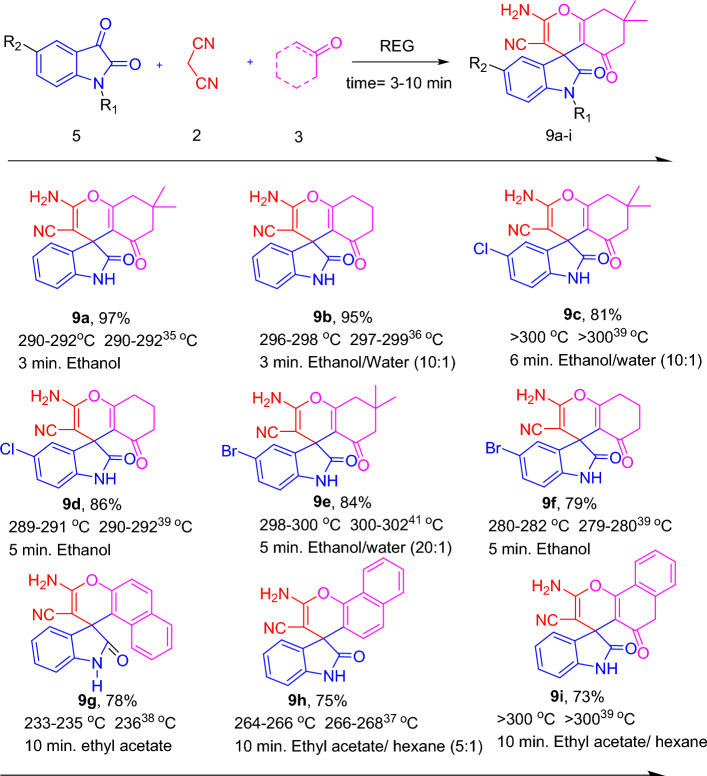
Synthesis of spiroxindole derivatives using REG. ^a^Yields refer to isolated pure product ^b^Crystallization Solvents.

In the continued exploration of the method, the REG system was employed for the green synthesis of different types of spirooxindoles (Fig. 6). This synthesis involved the use of isatin derivatives (5), malononitrile (2), and various types of Michael acceptors (3). By employing different combinations of these reactants, the selected multicomponent reaction (MCR) proceeded efficiently, resulting in the synthesis of the desired products with excellent yields within a short reaction time. The REG system, played a crucial role in facilitating this green synthesis process. By providing a suitable reaction environment, the REG system allowed for the efficient and rapid formation of spirooxindoles from the selected reactants. The utilization of the REG system in this context eliminated the need for additional catalysts or additives, emphasizing its utility as an environmentally friendly and efficient method for synthesis of various spirooxindole compounds.

Scalability holds immense importance in the industry due to the cost efficiency, the resilience and reliability of industrial systems. The scalability of the reaction was evaluated using the model reaction involving 4-chlorobenzaldehyde (**1a**), malononitrile (**2**), and dimedone (**3a**) on a 100 mmol scale. The reaction was successfully conducted on a larger scale, yielding the desired 4H-pyran derivatives with 97% isolated yields in just 50 min. However, it is important to note that precise control of reactant feeding is crucial for maintaining the temperature of the REG system within the desired range, preferably not exceeding room temperature. In larger-scale reactions, the feeding speed of reactants tends to be slower due to the need for careful monitoring and control. As a result, the overall reaction time slightly increase compared to smaller-scale reactions. Nonetheless, by ensuring proper control over the reactant feeding process, it is possible to maintain the desired temperature conditions and successfully carry out the reaction on a larger scale.

## Conclusion

To summarize, the study presents a highly efficient and additive-free method for synthesizing industrial-grade 2-amino-4-aryl-3-cyano-4H-chromenes, pyran, and spiroxindole derivatives using a mechanochemical approach. The method has distinct advantages over traditional ball mills, such as the ability to perform temperature-dependent reactions without external heating and its continuous operation, enabling easy scale-up. The heat generated during the reaction is a result of the speed of the rotating mortar and the friction between the conical part and the rotating mortar, making the process more energy-efficient. Overall, this approach represents a significant advancement in sustainable and efficient methods for organic synthesis.

## Experimental section

### Material and methods

In this study, the REG utilized for the experiments was custom-designed and manufactured within the laboratory conducting the research. The REG system was constructed using stainless steel 316 alloy for all its components. This choice of material ensures the system's durability and resistance to corrosion, which is important for maintaining the integrity of the equipment during the experimental process. The grinding frequency employed during the experiments using the REG system ranged between 15 and 20 Hz. This indicates the rate at which the grinding action took place within the system. The specific frequency chosen for the experiments depended on the optimization of the reaction conditions, considering factors such as the nature of the reactants, desired reaction kinetics, and the efficiency of mixing and grinding within the REG system. During the grinding process, it was observed that malononitrile, which has a melting point of 32 °C, undergoes melting when mixed with other reactants, resulting in a paste-like consistency. To determine the melting points of the compounds accurately, the Buchi B-545 Melting Point apparatus was employed.

For nuclear magnetic resonance (NMR) analysis, 500 MHz ^1^H NMR spectra were acquired using a Bruker DRX-500 Avance spectrometer. Additionally, 250 MHz spectra were obtained using a Bruker Avance DPX-250 spectrometer (Supplementary Information). In the experiments, solid reagents were obtained from commercial sources and used without additional purification. However, liquid reagents underwent distillation before being used. Thin-layer chromatography (TLC) was performed on silica gel plates using a 1:5 ratio of ethyl acetate to hexane as the eluent (Supplementary Information).

### Typical procedure for the synthesis of 2-amino-3-cyano-4-aryl-4H-chromenes

In a typical experiment, a stoichiometric mixture of an aromatic aldehyde (1) (5.0 mmol) or isatin derivatives (5) (5.0 mmol), malononitrile (2) (5.0 mmol), dimedone (3a) or other Michel acceptors (5.0 mmol) were introduced in REG system and grinded for the required duration (Fig. 3). Upon confirming the completion of the reaction using TLC, (in few cases only a single spot is observed on a TLC plate). The absence of solvents, catalysts, or additives in the reaction system simplifies the workup procedure, making it easier and more straightforward. The resulting precipitate obtained after the reaction was collected, and a recrystallization process was employed using the solvents illustrated in Figs. 4, 5, and 6 to further purify the product and obtain a highly pure compound. The synthesized products were previously known compounds^21–42^, and their identification and structural characterization were confirmed.

### Supplementary Information

Supplementary Information.

## Data Availability

The data that support the findings of this study are available on request from the corresponding author.

## References

[1] Achar TK, Bose A, Mal P (2017). Mechanochemical synthesis of small organic molecules. Beilstein J. Org. Chem..

[2] Bowmaker GA (2013). Solvent-assisted mechanochemistry. Chem. Commun..

[3] Tanaka K, Toda F (2000). Solvent-free organic synthesis. Chem. Rev..

[4] James SL, Adams CJ, Bolm C, Braga D, Collier P (2012). Mechanochemistry: Opportunities for new and cleaner synthesis. Chem. Soc. Rev..

[5] Crawford DE, Miskimmin CKG, Albadarin AB, Walker G, James SL (2017). Organic synthesis by Twin Screw Extrusion (TSE): Continuous, scalable and solvent-free. Green Chem..

[6] Crawford D, Casaban J, Haydon R, Giri N, McNally T, James SL (2015). Synthesis by extrusion: Continuous, large-scale preparation of MOFs using little or no solvent. Chem. Sci..

[7] Moad G (1999). The synthesis of polyolefin graft copolymers by reactive extrusion. Prog. Polym. Sci..

[8] Crawford, D. E., Wright, L. A., James, S.L., Abbott, A. P. Efficient continuous synthesis of high purity deep eutectic solvents by twin screw extrusion. *Chem. Comm*. **52**, 4215–4218 (2016). 10.1039/c5cc09685e 26911554

[9] Bonsignore L, Loy G, Secci D, Calignano A (1993). Synthesis and pharmacological activity of 2-oxo-(2H) 1-benzopyran-3-carboxamide derivatives. Eur. J. Med. Chem..

[10] Gao Y, Yang W, Du D-M (2012). Efficient organocatalytic asymmetric synthesis of 2-amino-4H-chromene-3-carbonitrile derivatives. Tetrahedron Asy..

[11] Ling R, Yoshida M, Mariano PS (1996). Exploratory investigations probing a preparatively versatile, pyridinium salt photoelectrocyclization-solvolytic aziridine ring opening sequence. J. Org. Chem..

[12] Kumar Krishnammagari S, Lim KT, Cho BG, Jeong YT (2018). Choline hydroxide: An efficient and biodegradable catalyst for the synthesis of 2-amino-3-nitro-4H-chromene derivatives in an aqueous medium. Phosphorus Sulfur Silicon Relat. Elem..

[13] Anamika Yadav CL, Drew MGB, Kumar K, Singh N (2021). Ferrocene-functionalized dithiocarbamate zinc(II) complexes as efficient bifunctional catalysts for the one-pot synthesis of chromene and imidazopyrimidine derivatives via Knoevenagel condensation reaction. Inorg. Chem..

[14] Zhang MZ, Chen Q, Yang GF (2015). A review on recent developments of indole-containing antiviral agents. Eur. J. Med. Chem..

[15] Kumari A, Singh RK (2019). Medicinal chemistry of indole derivatives: Current to future therapeutic prospectives. Bioorg. Chem..

[16] Wan Y, Li Y, Yan C, Yan M, Tang Z (2019). Indole: A privileged scaffold for the design of anti-cancer agents. Eur. J. Med. Chem..

[17] Taber DF, Tirunahari PK (2011). Indole synthesis: A review and proposed classification. Tetrahedron..

[18] Sebastião J, Neto S, Zeni G (2020). Recent advances in the synthesis of indoles from alkynes and nitrogen sources. Org. Chem. Front..

[19] Kaupp G, Naimi-Jamal MR, Schmeyers J (2003). Solvent-free Knoevenagel condensations and Michael additions in the solid state and in the melt with quantitative yield. Tetrahedron.

[20] Azizi N, Saidi MR (2013). Lithium perchlorate diethyl ether solution: A highly efficient media for the abramov reaction. Phosphorus Sulfur Silicon Relat. Elements.

[21] Auria-Luna F, Fernández-Moreira V, Marqués-López E, Gimeno MC, Herrera RP (2020). Ultrasound-assisted multicomponent synthesis of 4H-pyrans in water and DNA binding studies. Sci. Rep..

[22] Banerjee B, Priya A, Kaur M, Sharma A, Singh A, Gupta VK, Jaitak V (2023). Sodium dodecyl sulphate catalyzed one-pot three-component synthesis of structurally diverse 2-amino-3-*cyano substituted Tetrahydrobenzo[b]pyrans and spiropyrans in water at room temperature*. Catal. Lett..

[23] Naimi-Jamal MR, Mashkouri S, Sharifi A (2010). An efficient, multicomponent approach for solvent-free synthesis of 2-amino-4H-chromene scaffold. Mol. Divers..

[24] Guo RY, An ZM, Mo LP, Wang RZ, Liu HX, Wang SX, Zhang ZH (2013). Meglumine: A novel and efficient catalyst for one-pot, three-component combinatorial synthesis of functionalized 2-amino-4H-pyrans. ACS Comb. Sci..

[25] Nagaraju S, Paplal B, Sathish K, Giri S, Kashinath D (2017). Synthesis of functionalized chromene and spirochromenes using l-proline-melamine as highly efficient and recyclable homogeneous catalyst at room temperature. Tetrahedron Lett..

[26] Al-Omran F, El-Ghamry I, Elnagdi MH (1998). New spiropyran-4-ylindolidine derivatives from the reaction of 2-oxo-3-cyanomethylidene-2,3-dihydroindoles with cyclohexanediones and phenols. Org. Prep. Proced. Int..

[27] Shanthi G, Subbulakshmi G, Perumal PT (2007). A new InCl3-catalyzed, facile and efficient method for the synthesis of spirooxindoles under conventional and solvent-free microwave conditions. Tetrahedron.

[28] Wang GD, Zhang XN, Zhang ZH (2013). One-pot three-component synthesis of spirooxindoles catalyzed by hexamethylenetetramine in water. J. Heterocyclic Chem..

[29] Amirnejad M, Naimi-Jamal MR, Tourani H, Ghafuri H (2013). A facile solvent-free one-pot three-component method for the synthesis of 2-amino-4H-pyrans and tetrahydro-4H-chromenes at ambient temperature. Monatshefte fur Chemie.

[30] Balalaie S, Bararjanian M, Sheikh-Ahmadi M, Hekmat S, Salehi P (2007). Diammonium hydrogen phosphate: An efficient and versatile catalyst for the one-pot synthesis of tetrahydrobenzo[b]pyran derivatives in aqueous media. Synth. Commun..

[31] Hosseinchi O, Mashkouri S, Naimi-Jamal MR, Kaupp G (2014). Ball milling for the quantitative and specific solvent-free Knoevenagel condensation + Michael addition cascade in the synthesis of various 2-amino-4-aryl-3-cyano-4H-chromenes without heating. RSC Adv..

[32] Azizi N, Ahooie TS, Hashemi MM, Yavari I (2018). Magnetic graphitic carbon nitride-catalyzed highly efficient construction of functionalized 4 H-pyrans. Synlett.

[33] Khan AT, Lal M, Ali S, Khan MM (2011). One-pot three-component reaction for the synthesis of pyran annulated heterocyclic compounds using DMAP as a catalyst. Tetrahedron Lett..

[34] Shojaee S, Azizi N, Mirjafary Z, Saeidian H (2023). Magnet-responsive choline carbomer ionogels as a versatile and recyclable catalyst for one-pot synthesis of benzopyran in water. Sci. Rep..

[35] Xu J-C, Li W-M, Zheng H, Lai Y-F, Zhang P-F (2011). One-pot synthesis of tetrahydrochromene derivatives catalyzed by lipase. Tetrahedron.

[36] Zhang G, Zhang Y, Yan J, Chen R, Wang S, Ma Y, Wang R (2011). One-pot enantioselective synthesis of functionalized pyranocoumarins and 2-amino-4H-chromenes: Discovery of a type of potent antibacterial agent. J. Org. Chem..

[37] Azizi N, Dezfooli S, Khajeh M, Hashemi MM (2013). Efficient deep eutectic solvents catalyzed synthesis of pyran and benzopyran derivatives. J. Mol. Liq..

[38] Chen L, Bao S, Yang L, Zhang X, Li B, Li Y (2017). Cheap thiamine hydrochloride as efficient catalyst for synthesis of 4H-benzo[b]pyrans in aqueous ethanol. Res. Chem. Intermed..

[39] Mashkouri S, Naimi-Jamal MR (2009). Mechanochemical solvent-free and catalyst-free one-pot synthesis of pyrano[2,3-d]pyrimidine-2,4(1H,3H)-diones with quantitative yields. Molecules.

[40] Wang L-M, Jiao N, Qiu J, Yu J-J, Liu J-Q, Guo F-L, Liu Y (2010). Sodium stearate-catalyzed multicomponent reactions for efficient synthesis of spirooxindoles in aqueous micellar media. Tetrahedron.

[41] Dandia A, Parewa V, Jain AK, Rathore KS (2011). Step-economic, efficient, ZnS nanoparticle-catalyzed synthesis of spirooxindole derivatives in aqueous medium viaKnoevenagel condensation followed by Michael addition. Green Chem..

[42] Li Y, Chen H, Shi C, Shi D, Ji S (2010). Efficient one-pot synthesis of spirooxindole derivatives catalyzed by l-proline in aqueous medium. J. Comb. Chem..

