# Supplementary material for: Ultrafast and efficient continuous flow organic synthesis with a modified extruder-grinder system
Source: Sci Rep. 2024 Apr 27;14:9671. doi: 10.1038/s41598-024-59567-6 (PMC11053142; doi:10.1038/s41598-024-59567-6)
Supplement: Supplementary file 1 — Supplementary Information. [file 41598_2024_59567_MOESM1_ESM.pdf]

Ultrafast and Efficient Continuous Flow Organic Synthesis with a Modified Extruder-Grinder System

Omid Hosseinchi Qareaghaj, Mohammad Ghaffarzadeh, Najmedin Azizi*

Chemistry and Chemical Engineering Research Center of Iran, P.O. Box 14335-186, Tehran, Iran E-mail: azizi@ccerci.ac.ir

Advantages of REG over Ball-Mill

This continuous process allows for a more compact design as there is no need for batch processing or large holding tanks. The extruder-grinder-based system of REG enables a continuous flow of materials, which enhances efficiency and reduces the overall footprint of the equipment.

The reduced waste generation is another advantage of REG. Traditional batch processes often produce significant amounts of waste due to the need for multiple reaction vessels, cleaning procedures, and solvent disposal. In contrast, REG operates in a solvent-free and catalyst-free manner, minimizing waste generation and reducing the environmental impact of the synthesis process.

Energy savings are also a notable benefit of REG. The internal heat generation in the system eliminates the need for external heaters or the circulation of hot water or oil, which are common in some ball mill devices. By relying on frictional heat generation, REG reduces energy consumption, making it a more sustainable and cost-effective option for organic synthesis.

Moreover, REG offers time efficiency due to its continuous operation. Traditional batch processes often involve time-consuming steps such as loading and unloading of reaction vessels, heating and cooling cycles, and cleaning procedures. In contrast, REG's continuous flow system allows for a streamlined process, enabling faster synthesis and higher productivity.

The ability to experimentally control temperature through rotation frequency adjustments is another advantage of REG. This feature allows for precise temperature control, enhancing the reproducibility and scalability of the synthesis process. By optimizing the rotation frequency, researchers can achieve the desired temperature conditions for specific reactions, leading to improved reaction outcomes and product quality.

In summary, REG, the novel extruder-grinder-based continuous system for organic synthesis, offers several advantages over traditional batch processes and even some ball mill devices. These advantages include reduced waste, energy savings, time efficiency, independence from external heat sources, smaller size compared to industrial ball mills (Figure S1), and the ability to experimentally control temperature through rotation frequency adjustments. These features make REG a promising option for solvent-free and catalyst-free green synthesis in the chemical and pharmaceutical industries, addressing the need for more sustainable and efficient manufacturing processes.

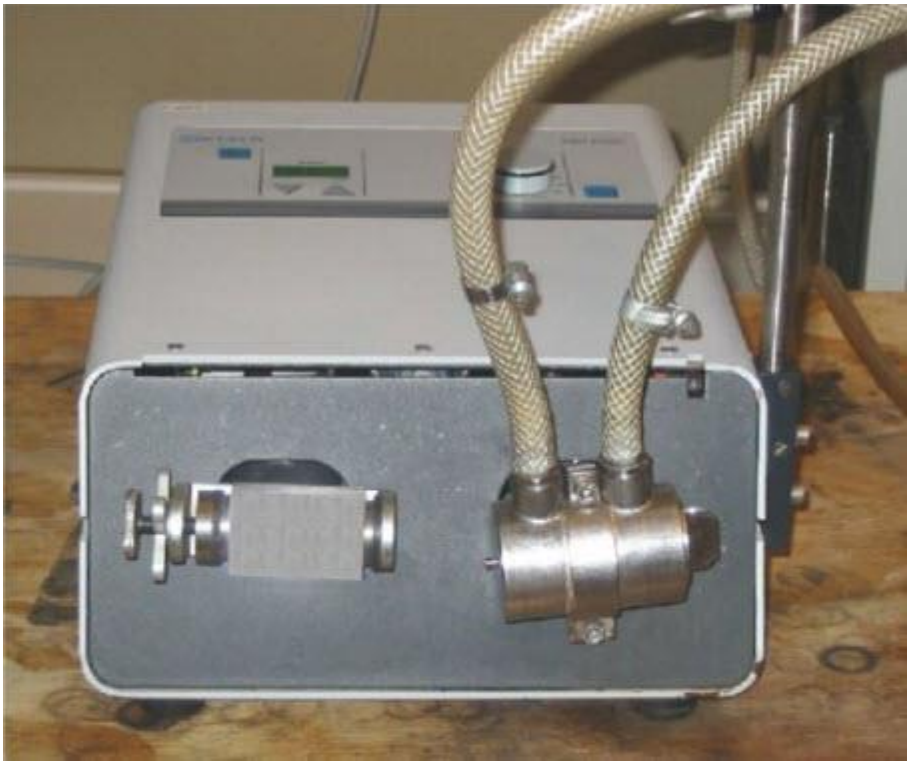

Figure S1. Classical Ball-mill used in organic synthesis

Experimental Section

Material and methods

In this study, the REG utilized for the experiments was custom-designed and manufactured within the laboratory conducting the research. The REG system was constructed using stainless steel 316 alloy for all its components. This choice of material ensures the system's durability and resistance to corrosion, which is important for maintaining the integrity of the equipment during the experimental process. The grinding frequency employed during the experiments using the REG system ranged between 15 to 20 Hz. This indicates the rate at which the grinding action took place within the system. The specific frequency chosen for the experiments depended on the optimization of the reaction conditions, considering factors such as the nature of the reactants, desired reaction kinetics, and the efficiency of mixing and grinding within the REG system.

During the grinding process, it was observed that malononitrile, which has a melting point of 32°C, undergoes melting when mixed with other reactants, resulting in a paste-like consistency. To determine the melting points of the compounds accurately, a Barnstead electrothermal melting point apparatus was employed. This apparatus is specifically designed for measuring and controlling the temperature at which compounds undergo the transition from a solid to a liquid state.

Infrared (IR) spectra were recorded using a Shimadzu 8400s FT-IR spectrometer. The samples were prepared as potassium bromide (KBr) pellets, which serve as the matrix for the analysis. The FT-IR spectrometer measures the absorption of infrared light by the sample, providing information about the

functional groups present in the molecules. For nuclear magnetic resonance (NMR) analysis, 500 MHz 1H NMR spectra were acquired using a Bruker DRX-500 Avance spectrometer. Additionally, 250 MHz spectra were obtained using a Bruker Avance DPX-250 spectrometer.

In the experiments, solid reagents were obtained from commercial sources and used without additional purification. However, liquid reagents underwent distillation before being used. The synthesized products were previously known compounds, and their identification and structural characterization were confirmed. The physical and spectral data obtained for the products were consistent with the corresponding literature reports. To assess the purity of the products, several methods were employed. Melting point determination was carried out to check if the observed melting points matched the reported values. Thin-layer chromatography (TLC) was performed on silica gel plates using a 1:5 ratio of ethyl acetate to hexane as the eluent. This technique allows for the visualization of different compounds in the product mixture. Additionally, ^{1}H NMR spectra were used to analyze the chemical shifts and coupling patterns, providing further confirmation of the product's identity and purity.

Typical procedure for the synthesis of 2-amino-3-cyano-4-aryl-4H-chromenes:

In a typical experiment, a stoichiometric mixture of an aromatic aldehyde (1) (5.0 mmol) or isatin derivatives (5) (5.0 mmol), malononitrile (2) (5.0 mmol), dimedone (3a) or other Michel acceptors (5.0 mmol) were introduced in REG system and grinded for the required duration (Figure 3). Upon confirming the completion of the reaction through analytical techniques such as FT-IR or TLC analysis, the resulting precipitate was treated with recrystallization either ethanol, methanol or ethyl acetate as the solvent to obtain highly pure products.

Selected compounds:

(4e):

(FTIR): ν = 3405, 3325, 3182, 3180, 2968, 2190, 1683, 1518, 1367 cm^{-1}. ^{1}H NMR (500 MHz, DMSO-d_6) δ = 1.017 (s, 3H, CH_3), 1.035 (s, 3H, CH_3), 2.019–2.139 (dd, 2H), 2.499–2.626 (d, 2H), 4.164 (s, 1H), 7.628–7.655 (d, 2H), 8.254–8.280 (d, 2H). EI-MS *m/z* (%): 339 (M^+, 1), 338 (5), 325 (2), 122 (54), 216 (5), 45 (11), 293 (100).

(7b):

(FTIR): ν = 3452, 3337, 2181, 1664, 1601, 1570, 1402 cm^{-1}. ^{1}H NMR (500 MHz, DMSO-d_6) δ = 5.170 (s, 1H, CH), 6.628–6.643 (d, 1H), 6.885 (s, 2H), 6.974–6.989 (d, 2H), 7.307–7.325 (d, 1H), 7.416–7.449 (m, 1H), 7.842–7.858 (d, 1H), 7.905-7.916 (d, 2H). EI-MS *m/z* (%): 376 (M^+, 22), 338 (M^{+2}, 25), 154 (32), 375 (31), 142 (16), 221 (100), 299 (15).

(7c):

(FTIR): ν = 3464, 3357, 3206, 2193, 1658, 1589, 1411 cm^{-1}. ^{1}H NMR (500 MHz, DMSO-d_6) δ = 5.632 (s, 1H, CH), 7.385–7.403 (d, 1H), 7.420–7.483 (m, 2H), 7.559–7.601 (t, 1H), 7.658–7.683 (d, 1H), 7.853–7.883 (d, 1H), 7.932–7.947 (d, 1H), 7.983-8.001 (d, 1H), 8.038-8.054 (d, 1H), 8.081 (s, 1H). EI-MS *m/z* (%): 343 (M^+, 1), 342 (4), 297 (6), 122 (52), 221 (7), 126 (12), 46 (100).

(9a):

(FTIR): ν = 3313, 3144, 2961, 2193, 1725, 1682, 1656, 1471, 1350, 1055, 765, 617 cm^{-1}. ^{1}H NMR (500 MHz, DMSO-d_6) δ = 1.026 (s, 3H, CH_3), 1.058 (s, 3H, CH_3), 2.160–2.202 (m, 2H), 2.511–2.614 (m, 2H), 6.971 (m, 1H), 7.110–7.138 (m, 1H), 7.299–7.319 (m, 1H), 7.489 (m, 1H), 10.349 (s, 1H). EI-MS *m/z* (%): 335 (M^+, 1), 334 (13), 15 (14), 131 (75), 269 (5), 333 (10), 320 (100).

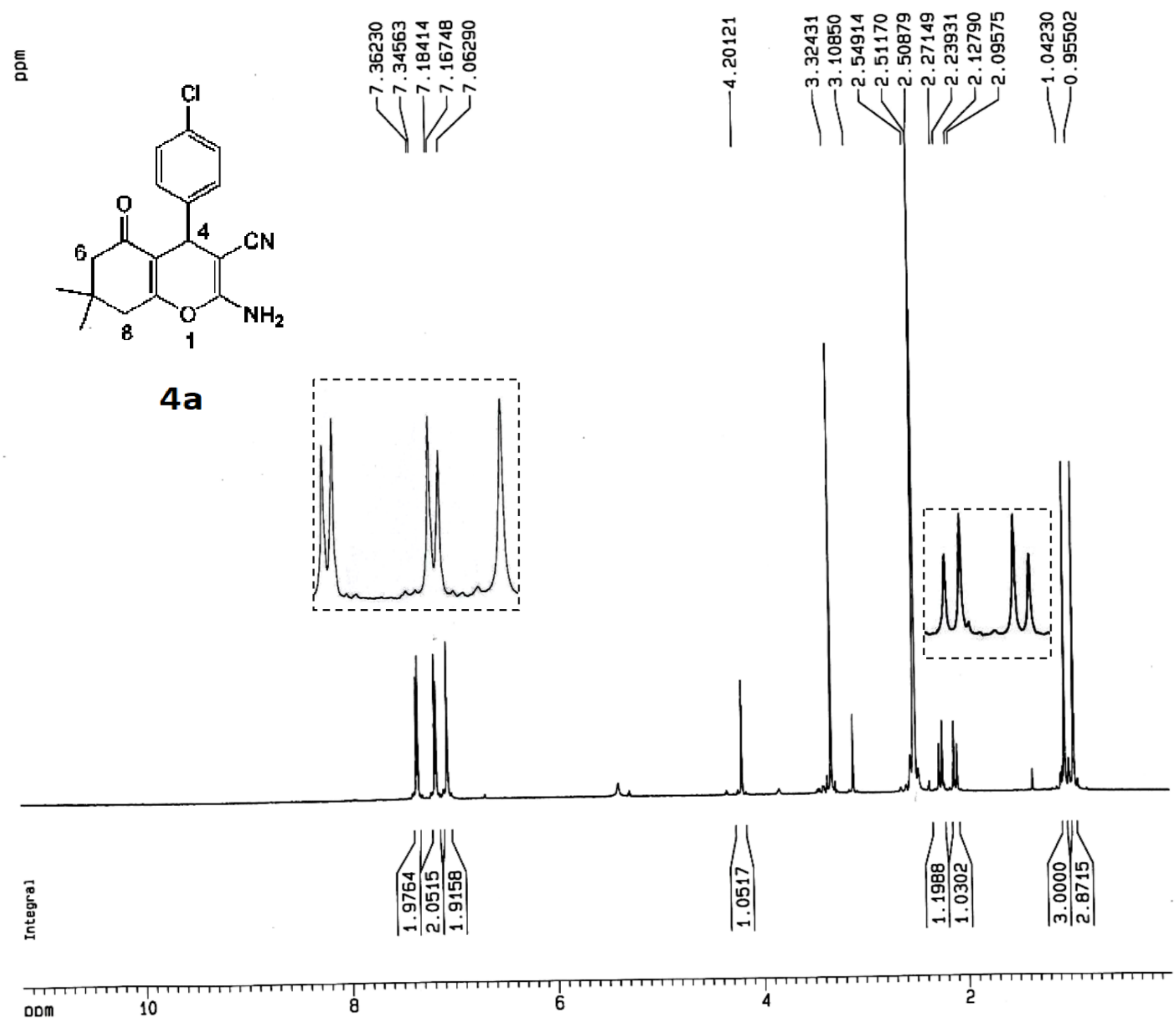

Figure S1: ^{1}H NMR Spectra of compound **4a**

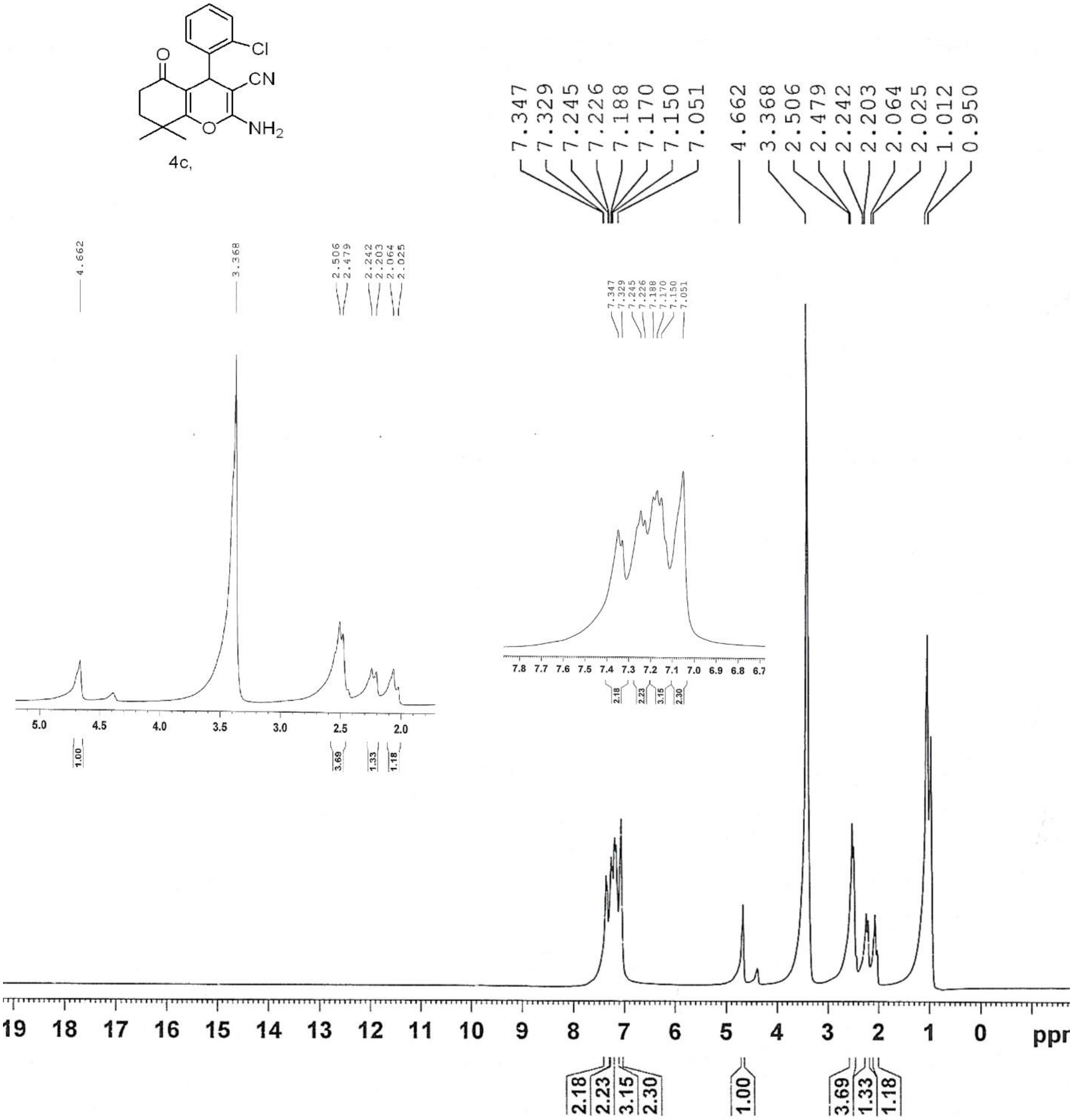

Figure S2: ^{1}H NMR Spectra of compound **4c**

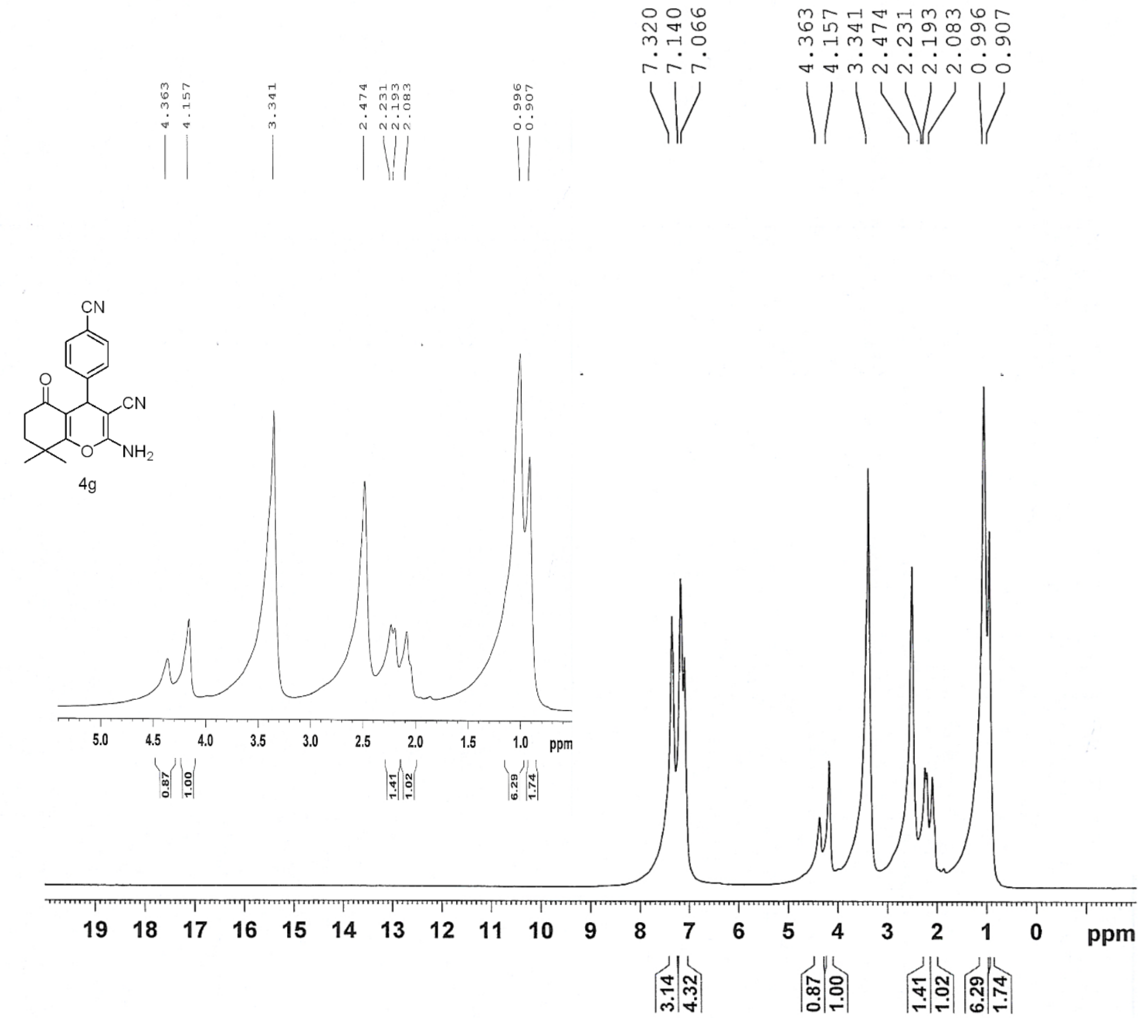

Figure S3: ^{1}H NMR Spectra of compound **4g**

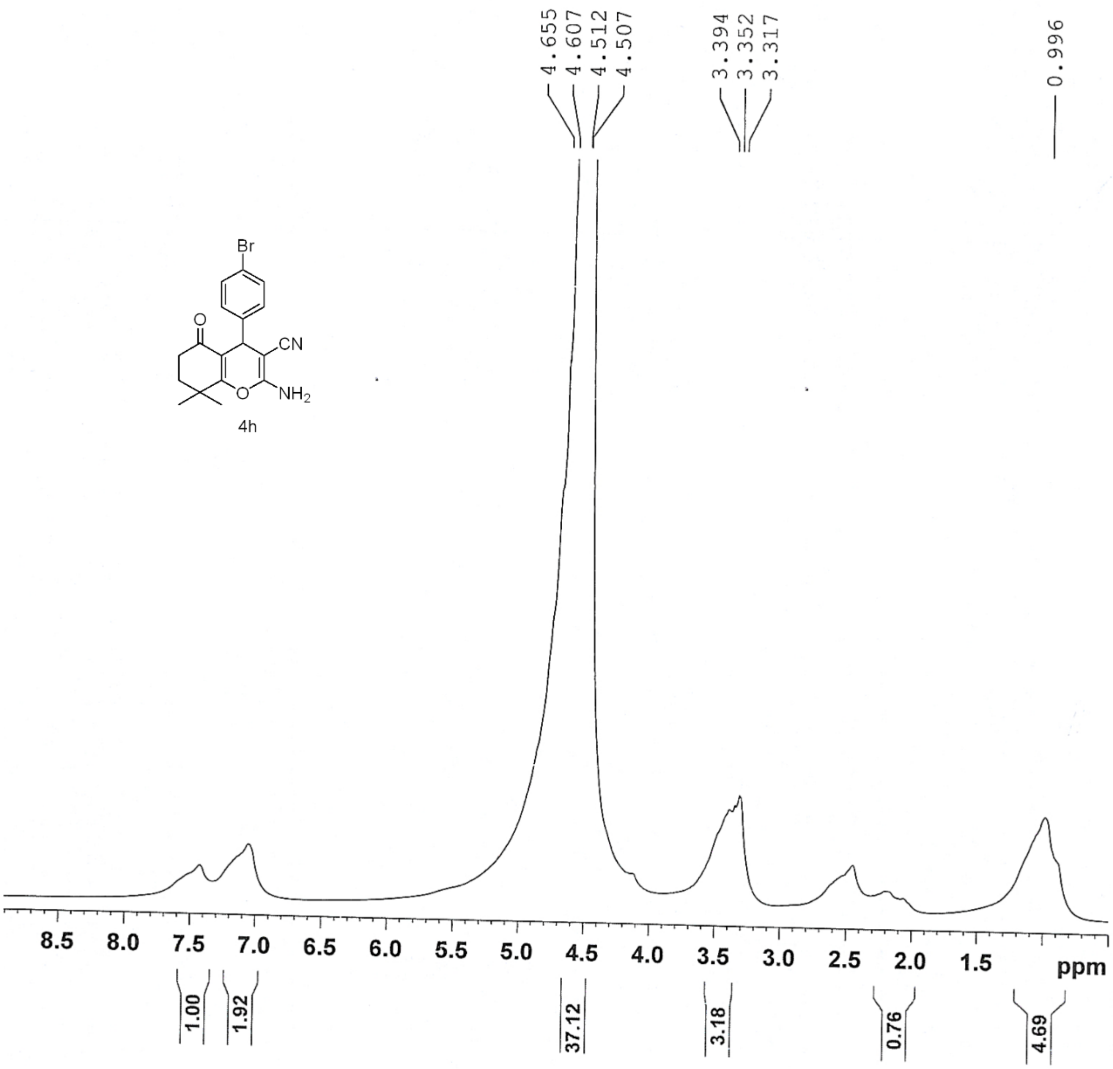

Figure S4: ^{1}H NMR Spectra of compound 4h

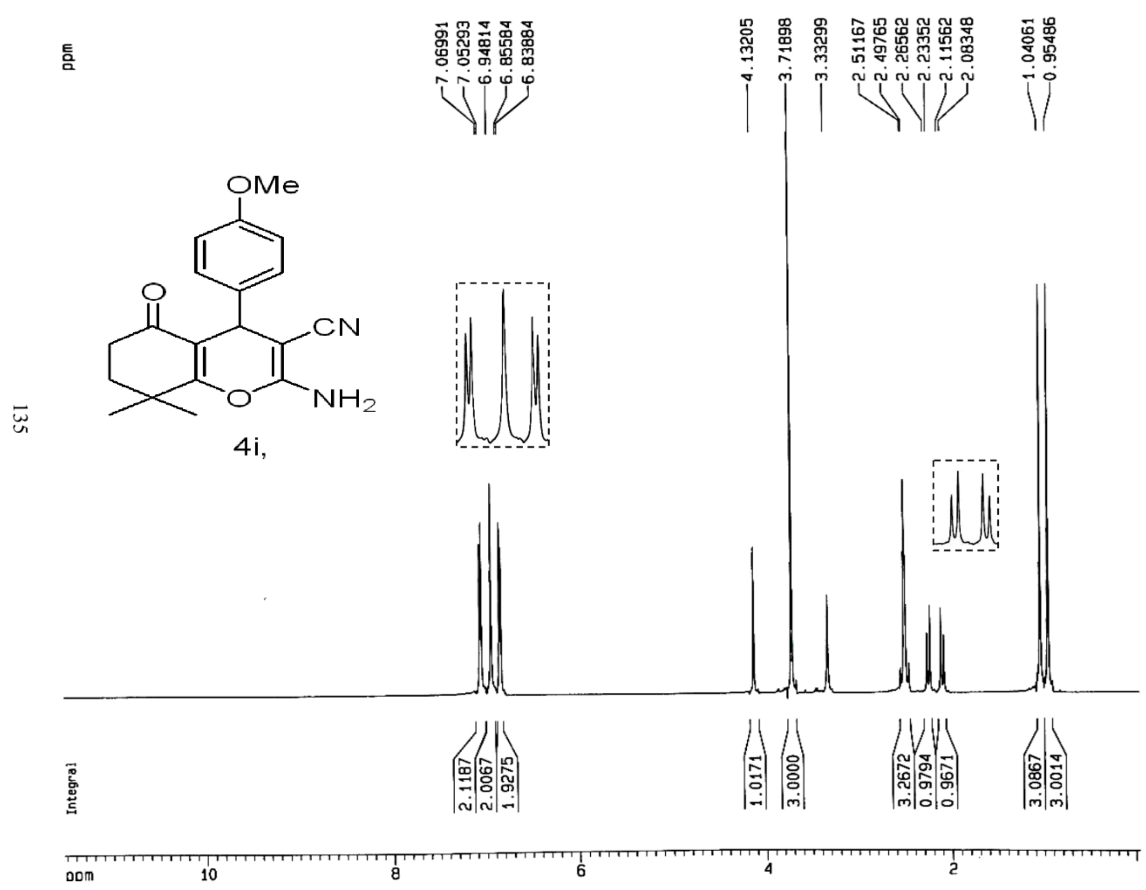

Figure S5: ^{1}H NMR Spectra of compound **4i**

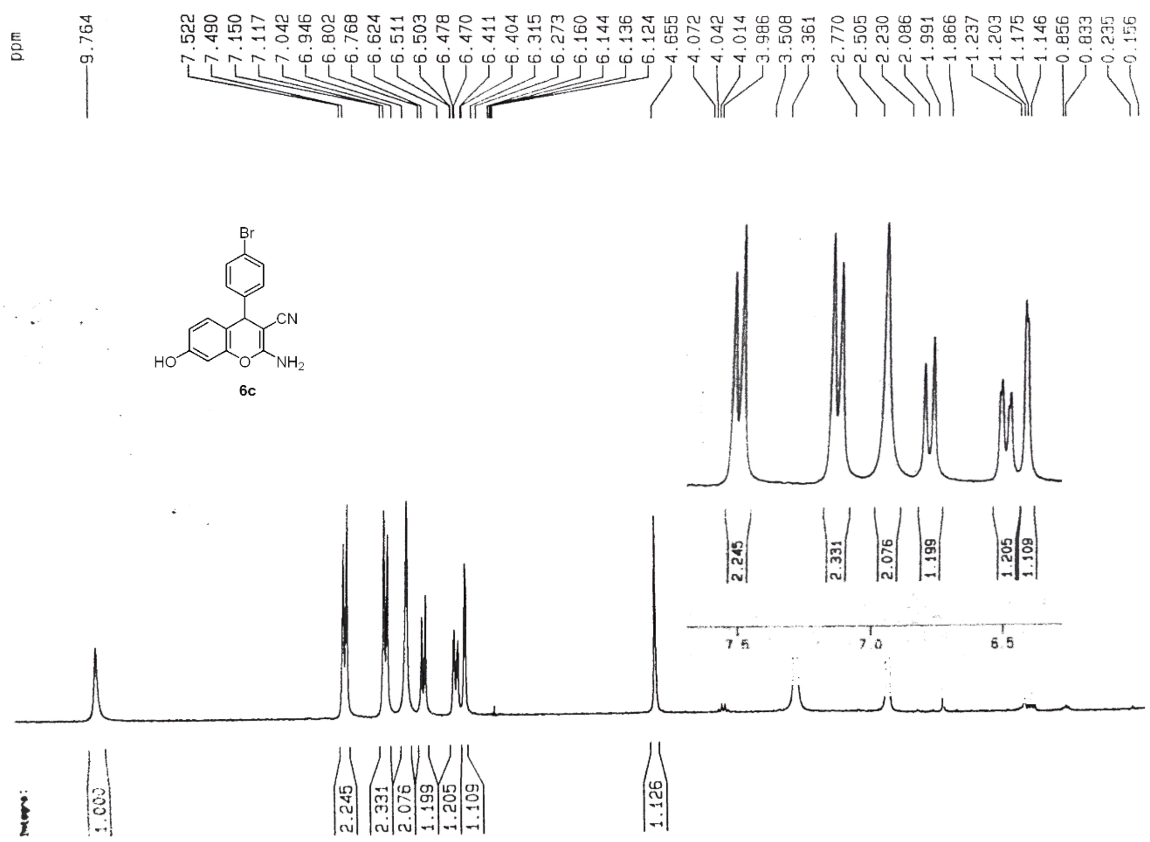

Figure S6: ^{1}H NMR Spectra of compound **6c**

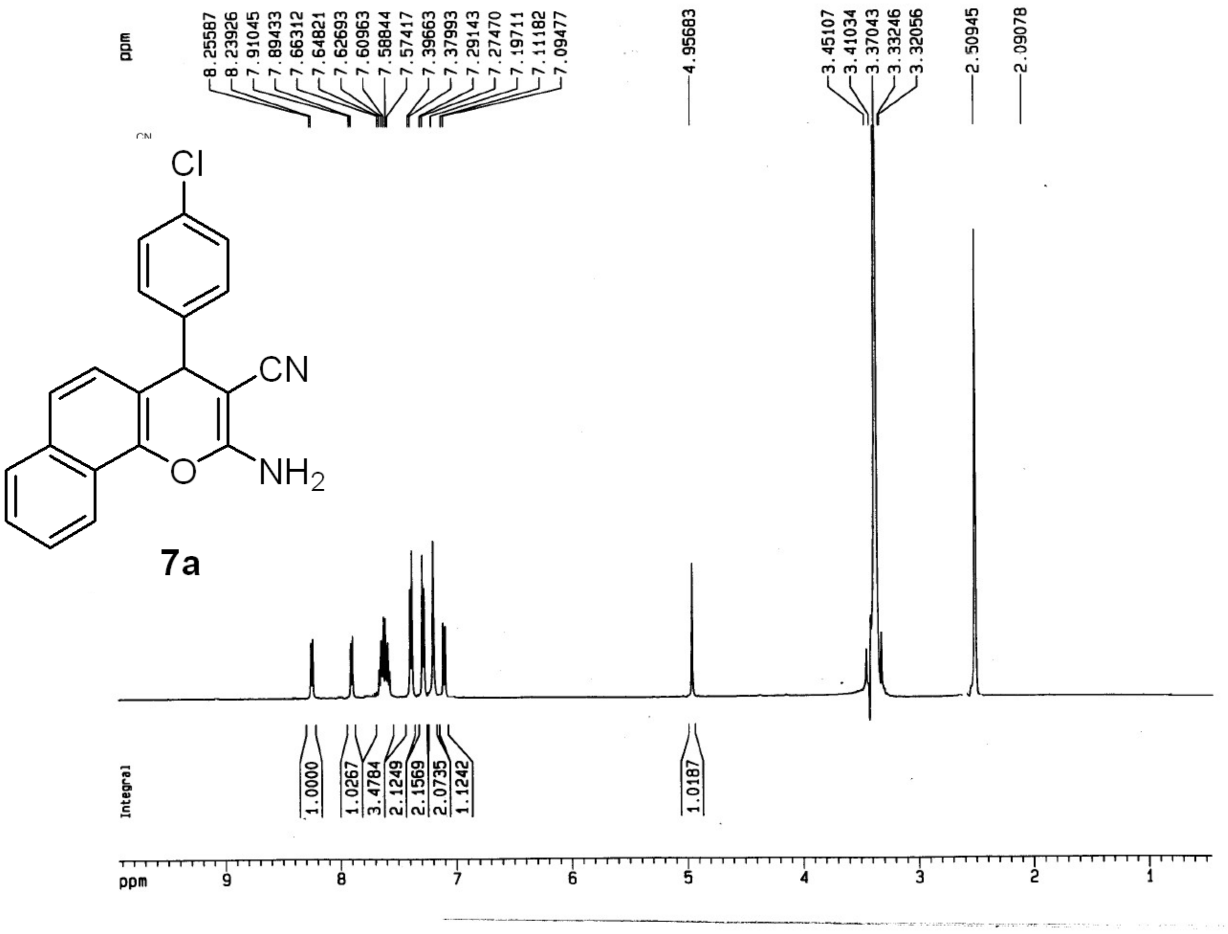

Figure S7: ^{1}H NMR Spectra of compound **7a**

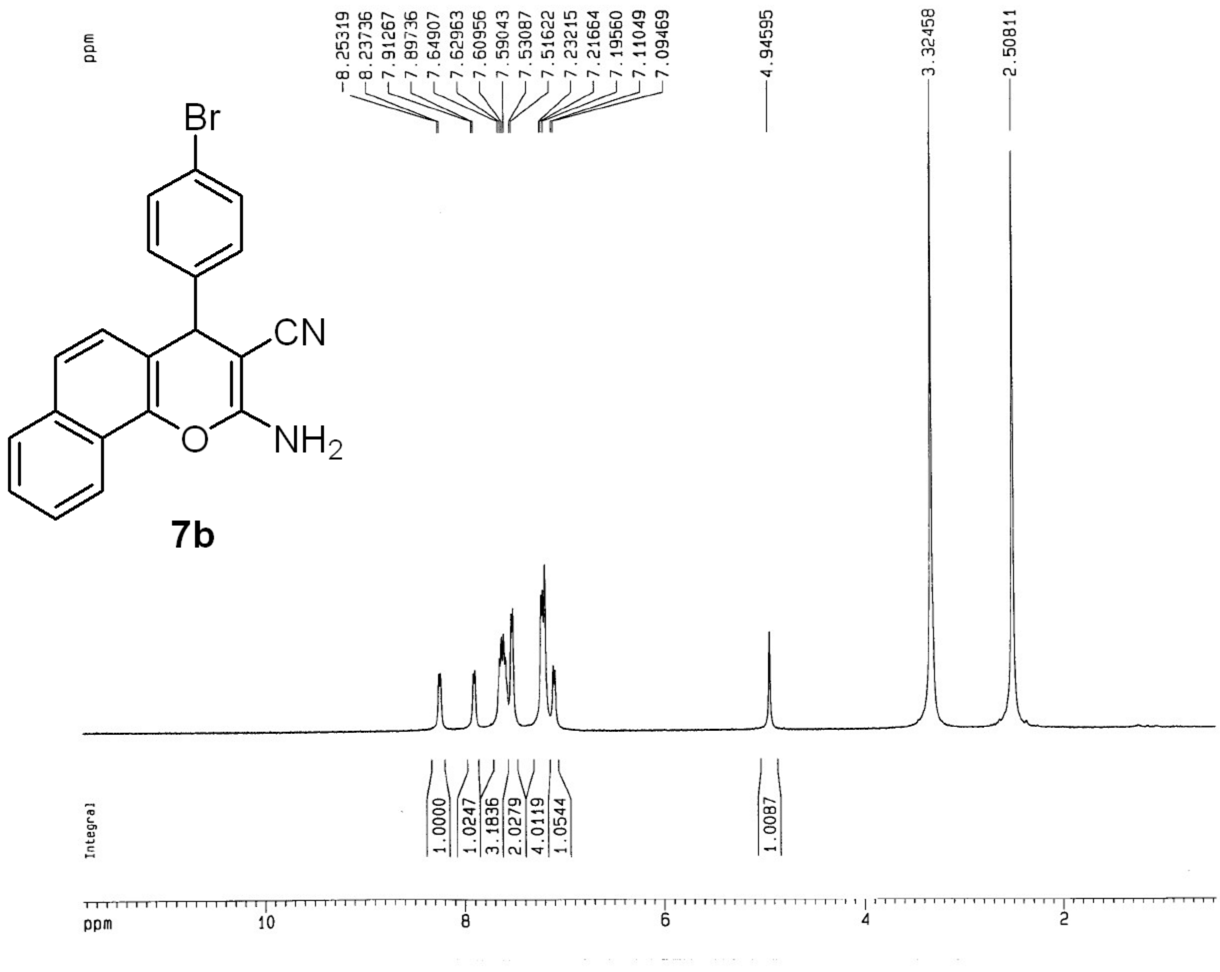

Figure S8: ^{1}H NMR Spectra of compound **7b**

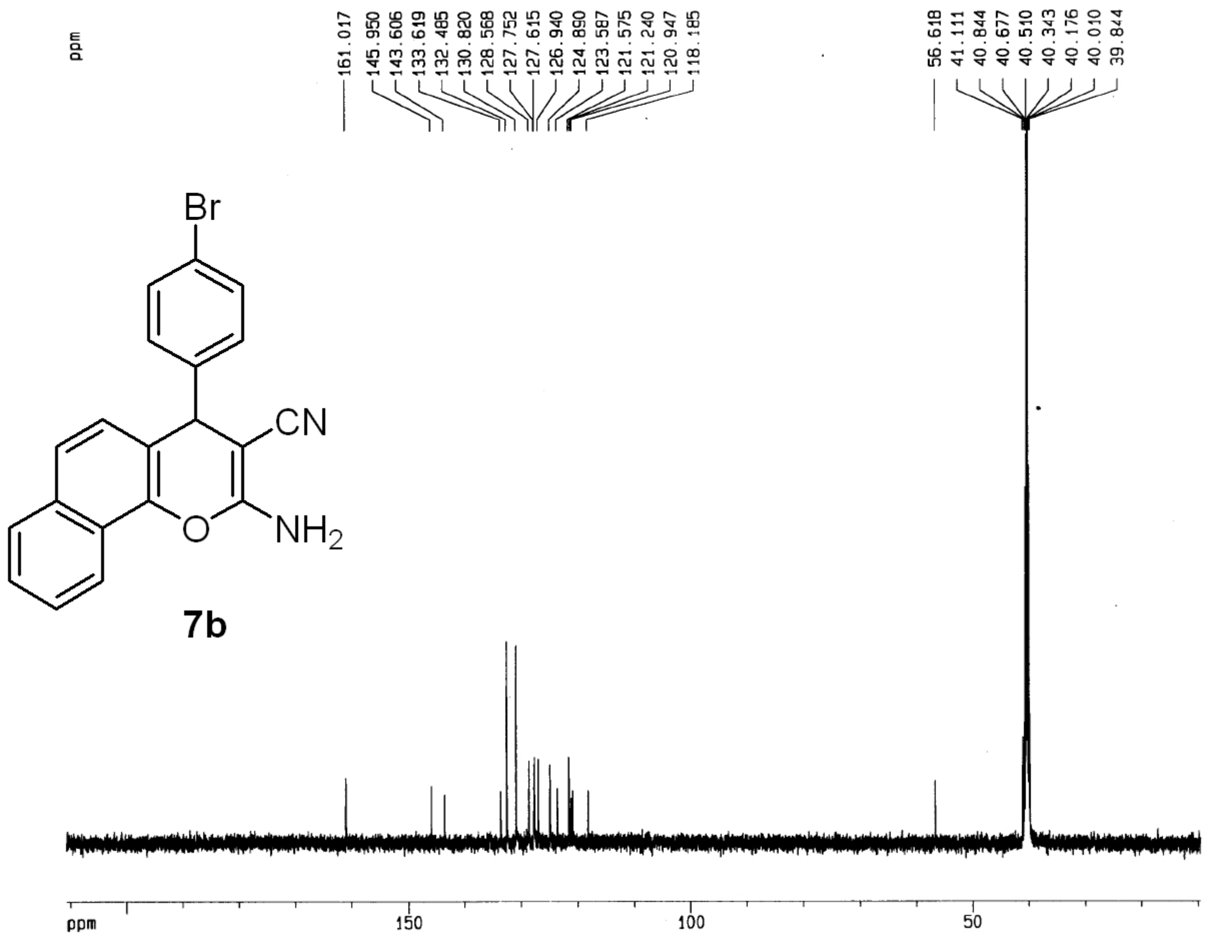

Figure S9: ^{13}C NMR Spectra of compound **7b**

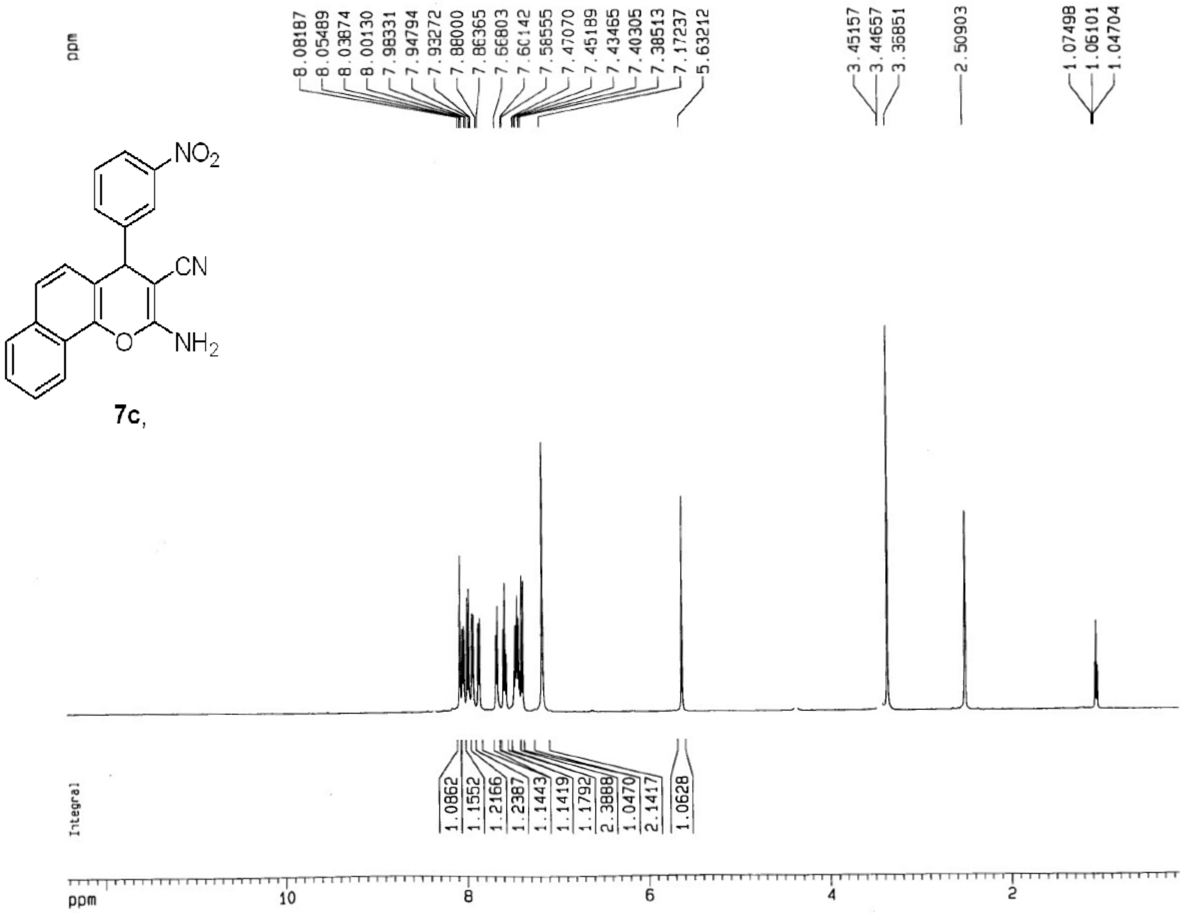

7c,

Figure S10: ^{1}H NMR Spectra of compound **7c**

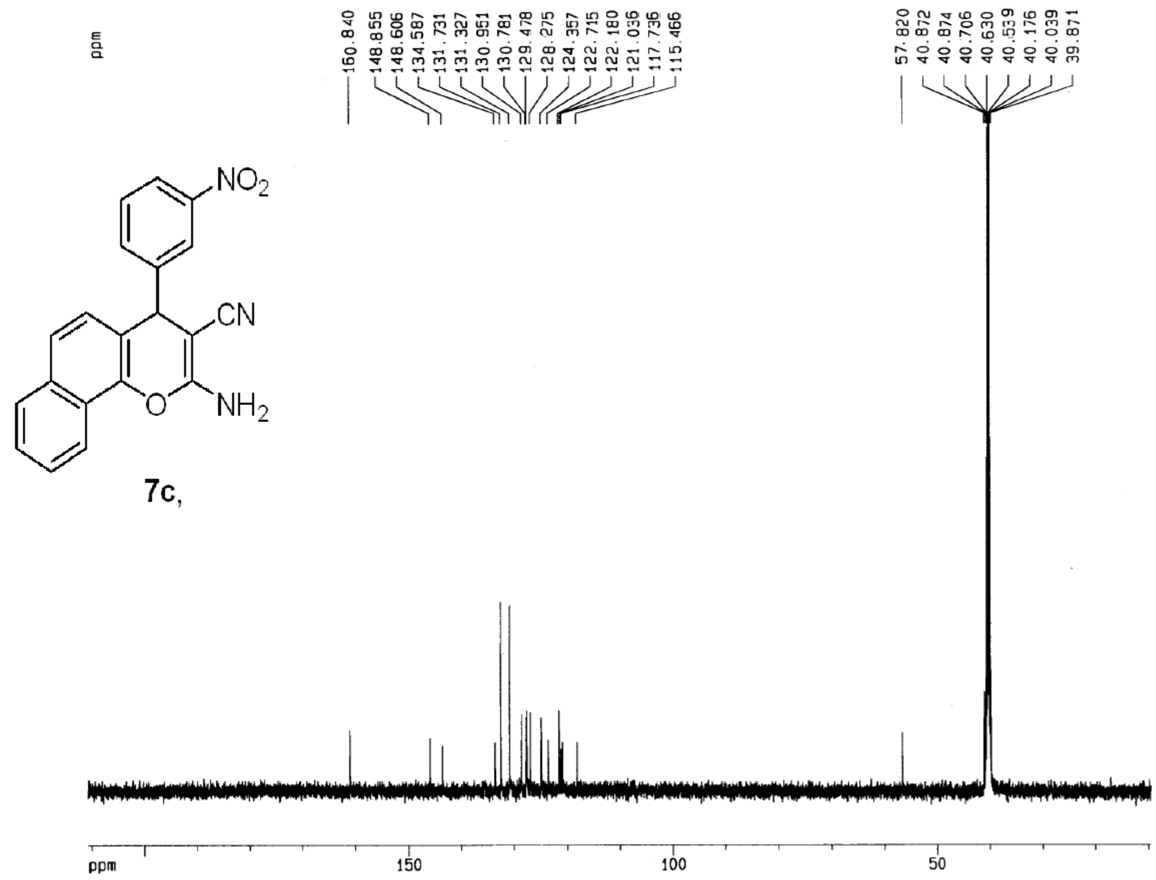

Figure S11: ^{13}C NMR Spectra of compound **7c**

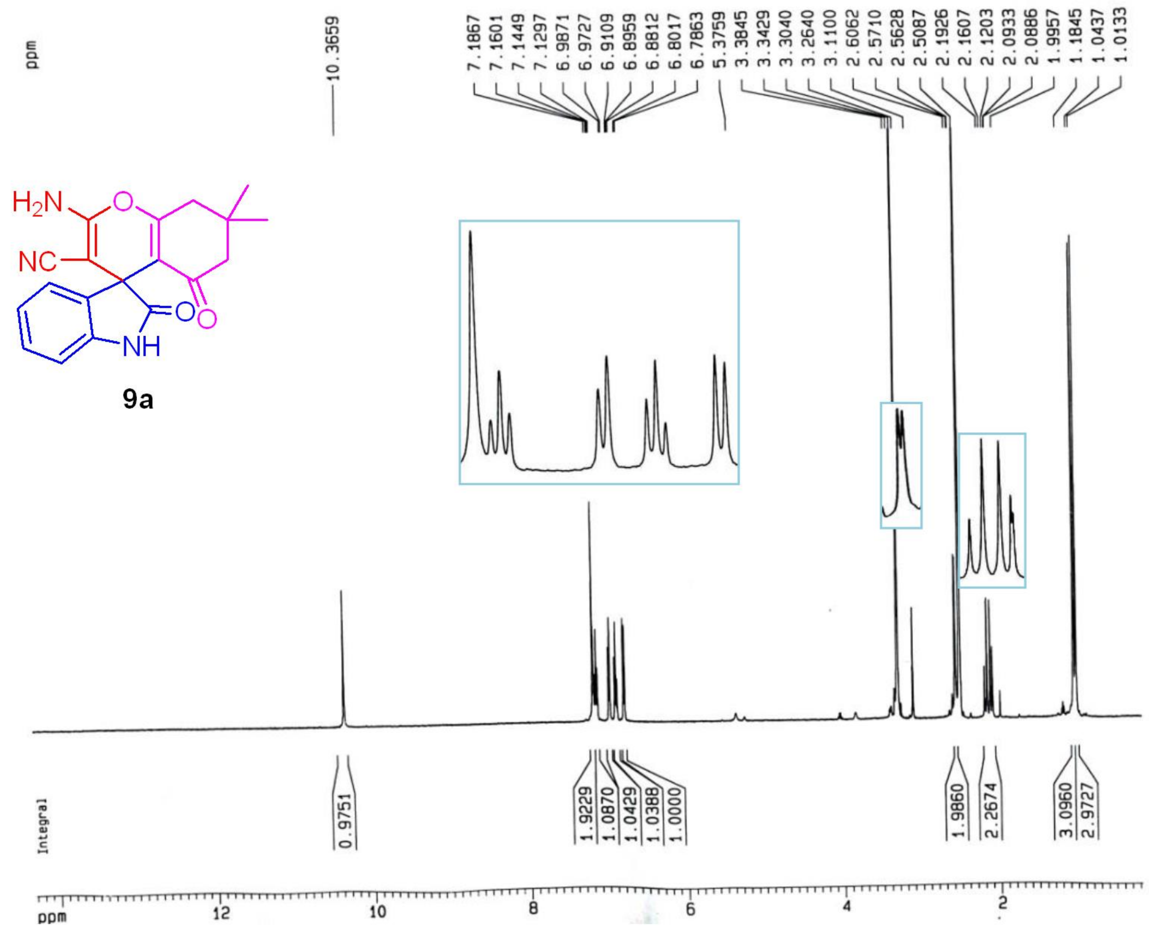

Figure S12: ^{1}H NMR Spectra of compound **9a**

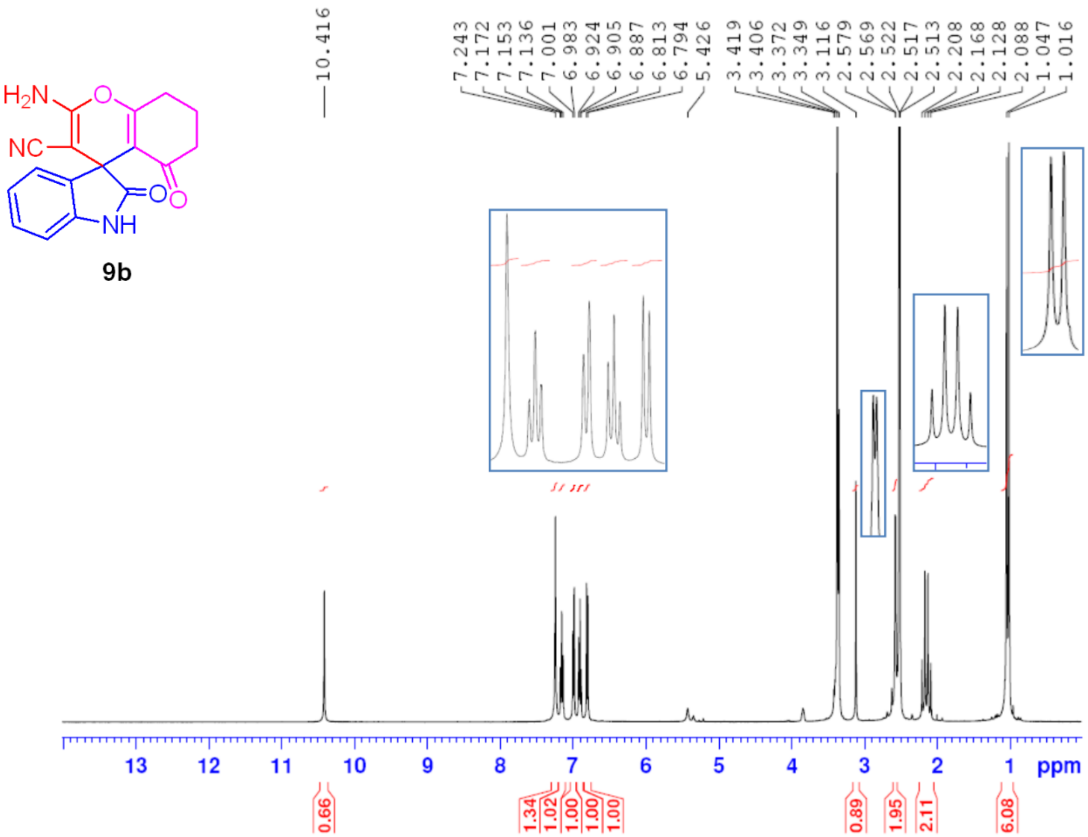

Figure S13: ^{1}H NMR Spectra of compound **9b**

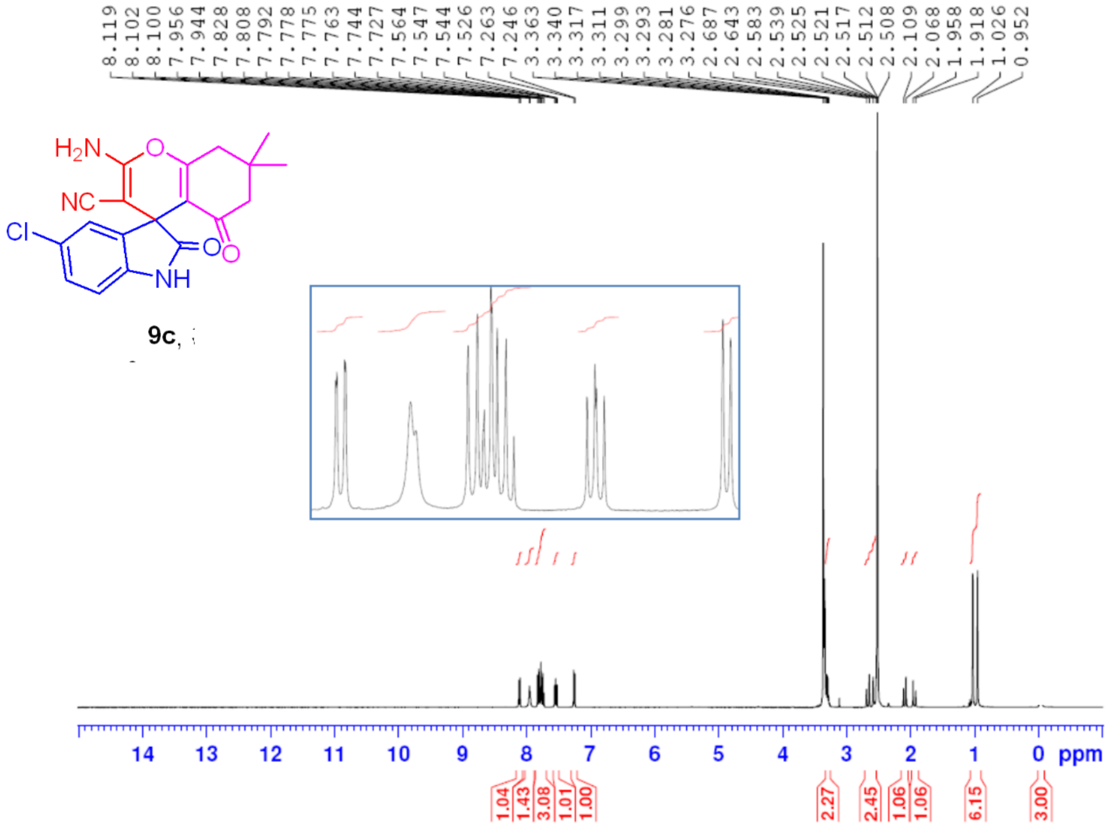

Figure S14: ^{1}H NMR Spectra of compound **9c**

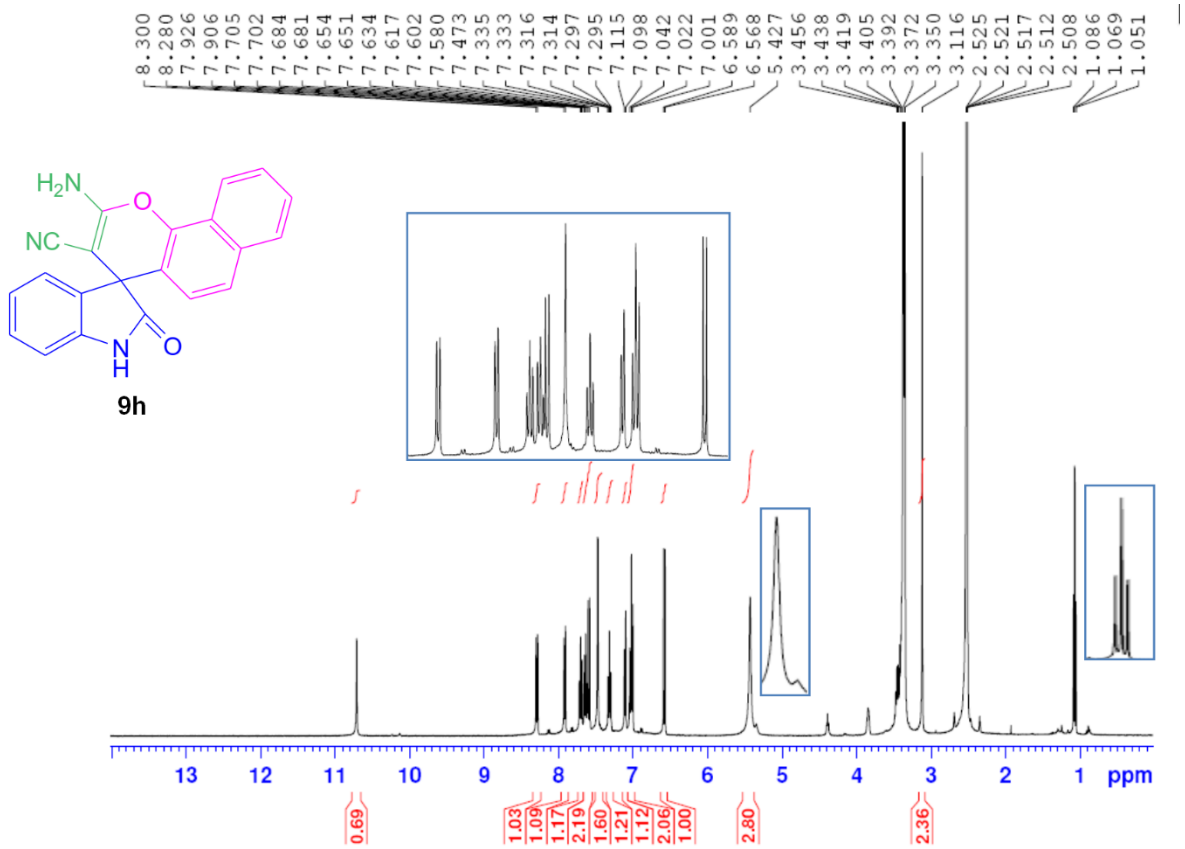

Figure S15: ^{1}H NMR Spectra of compound **9h**

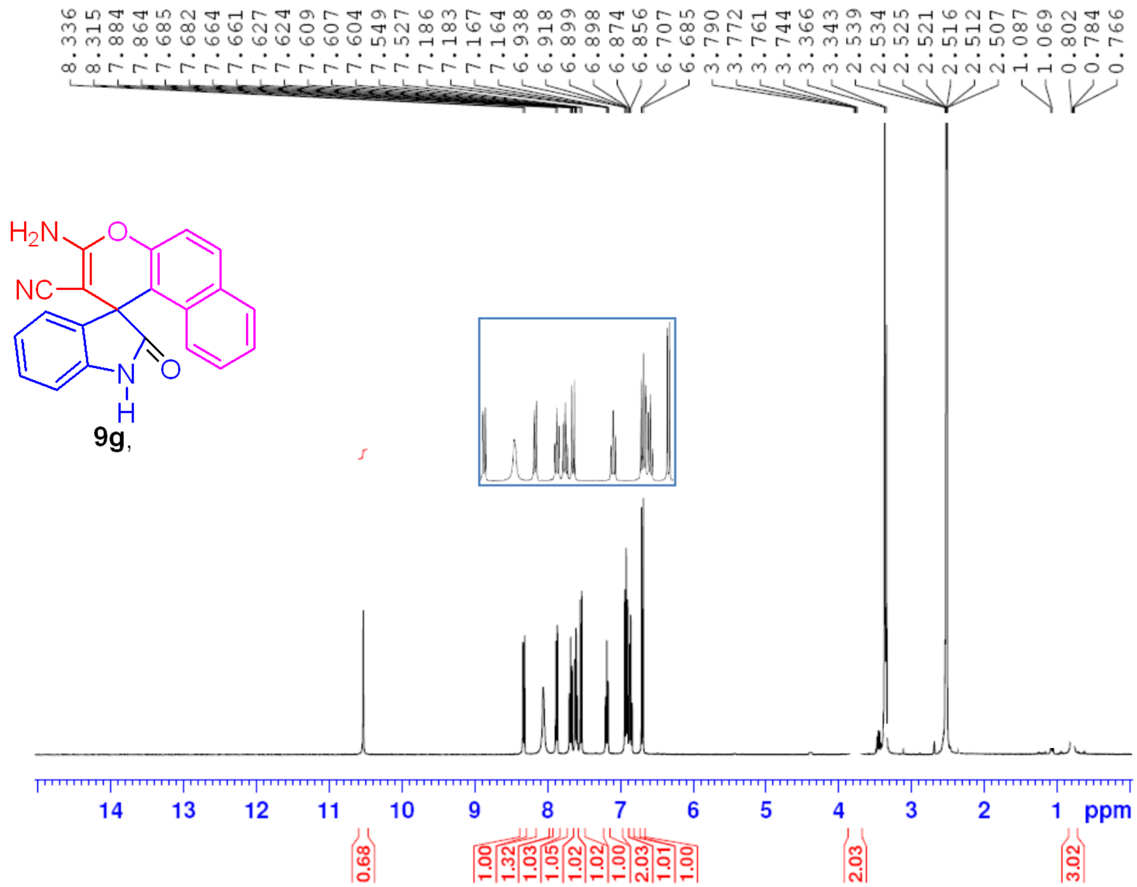

Figure S16: ^{1}H NMR Spectra of compound **9g**

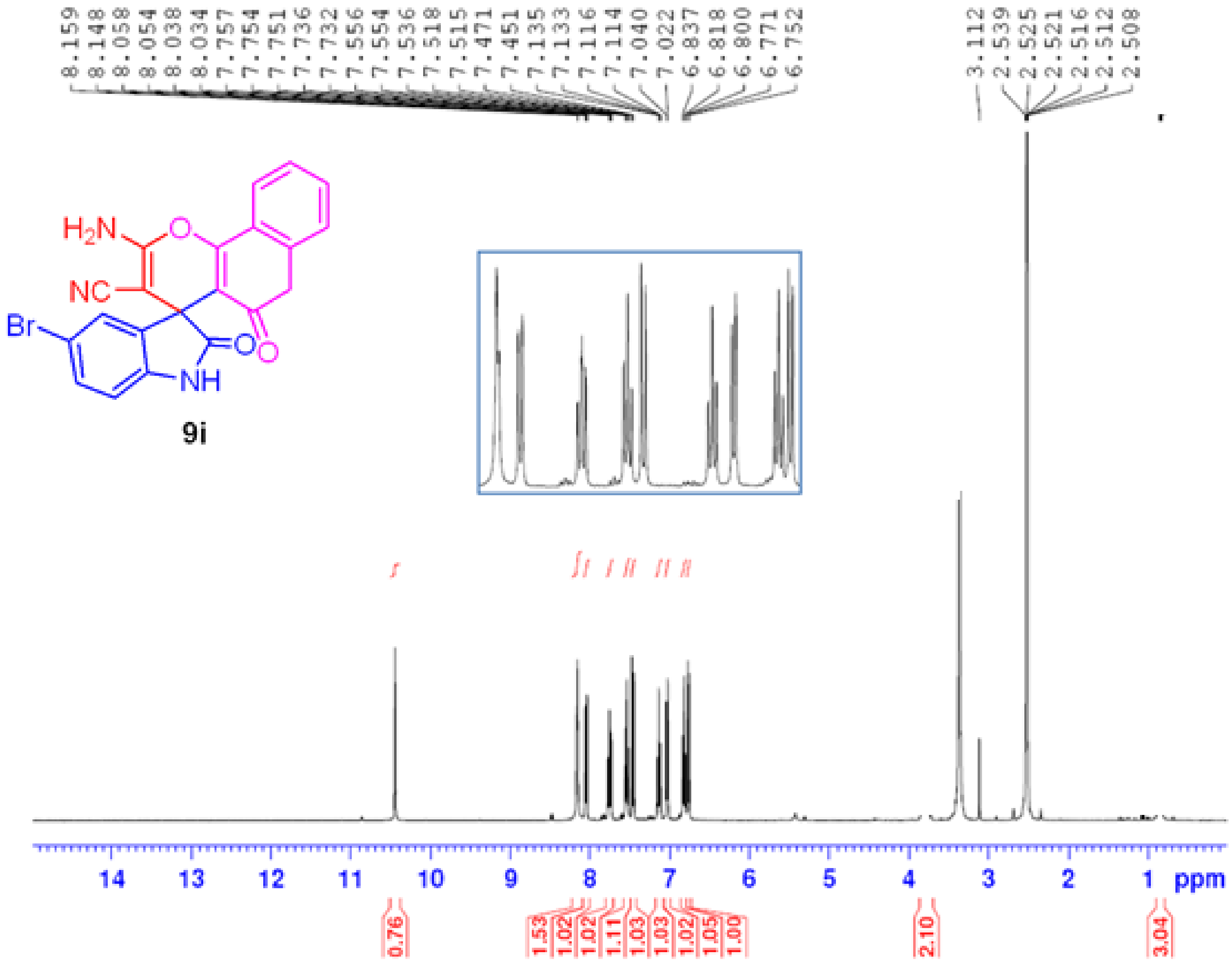

Figure S17: ^{1}H NMR Spectra of compound **9i**
